# Perfusion pressure as a determinant of respiratory function outcomes in unilateral biportal lumbar endoscopic procedures

**DOI:** 10.3389/fphar.2025.1593118

**Published:** 2025-05-30

**Authors:** Liang Zhang, Han Zheng, Yan Fu, Wenbo Li, Jianlong Lang, Yi Wang, Weibin Ren

**Affiliations:** ^1^ Department of Orthopedics, The Second Hospital of Tianjin Medical University, Tianjin, China; ^2^ Department of Radiology The Second Hospital of Tianjin Medical University, Tianjin, China; ^3^ First Teaching Hospital of Tianjin University of Traditional Chinese Medicine, National Clinical Research Center for Chinese Medicine Acupuncture and Moxibustion, Tianjin, China; ^4^ Rehabilitation Medicine Department, Binhai Hospital of Tianjin Medical University General Hospital, Tianjin, China; ^5^ Department of Pain Management, Zhongnan Hospital of Wuhan University, Wuhan, China; ^6^ Department of Orthopedics, Shexian Hospital, Shexian, Hebei, China

**Keywords:** unilateral biportal endoscopy, diaphragm electromyography, respiratory movement, perfusion water pressure, lumbar vertebra

## Abstract

**Introduction:**

UBE is used to treat most lumbar spine diseases, and it must rely on continuous infusion of saline to maintain a clear field of vision during the operation to ensure the smooth progress of the operation. Among many complications, the incidence of dural tear is the highest. Whether UBE can damage the dura and the effect of intraoperative perfusion pressure changes on respiratory function under different conditions are not clear.

**Methods:**

In the present experiment, Wistar rats were implanted with diaphragmatic electrodes and divided into two groups (dura mater with rupture group and dura mater without rupture group). In the experiment, the perfusion pressure was continuously increased, and the water pressure was 6KPa, 10KPa and 14Kpa for 2 min, respectively. The changes of respiratory movement were observed and analyzed. The preoperative and postoperative MRI scan results were compared. Pathological staining was used to observe the spinal cord injury.

**Results:**

Finally, we found that high perfusion pressure impaired respiratory function in rats with dural rupture, mainly manifested as decreased respiratory rate, but had no significant effect on respiratory function in rats with intact dura mater. HE staining and toluidine blue staining showed more nishi in the cauda equina nerve of the rats in the dural rupture group. Immunofluorescence results showed that the degree of cauda equina nerve injury in the dural rupture group was more severe than that in the dural rupture group.

**Discussion:**

This study reveals the effects of perfusion pressure and dural injury on respiratory function in UBE, and avoiding dural sac rupture is an effective means to prevent and treat complications of UBE, which will provide a new perspective on UBE.

## Introduction

In recent years, unilateral dual-channel technology has been rapidly developed and improved in spinal surgery. During the operation, the patient is kept in the prone position, and during the operation, plasma radiofrequency ablation equipment is applied to deal with the soft tissues. The indications for the procedure have gradually expanded from disc herniation to spinal stenosis, foraminal stenosis, and the procedure has been officially named UBE (unilateral biportal endoscopy). The process of its development in recent years has been broadly as follows. In 2013, a discectomy was performed using the UBE technique (Soliman, 2013). In 2016, the UBE technique was used for the treatment of lumbar spinal stenosis and named biportal endoscopic spinal surgery (BESS). In 2017, a total endoscopic spinal fusion was performed using the UBE technique (Heo et al., 2017). In 2010, a total endoscopic spinal fusion was performed using the UBE technique. total endoscopic spinal fusion (Akbary et al., 2018). In 2018, nerve root decompression was performed using a contralateral approach (Akbary et al., 2018). In 2018, patients with L5-S1 foraminal stenosis were decompressed using a 30° arthroscope and lumbar interbody fusion was performed using an intervertebral foraminal approach (Kim and Choi, 2018). In 2019, patients with compression of the L5 nerve root due to an extra vertebral foraminal air-containing pseudocyst were treated using the UBE technique (An and Lee, 2019).In 2010, patients with L5 nerve root compression due to an extra vertebral foraminal air-containing pseudocyst were treated using the BESS technique. In 2020, unilateral laminotomy with bilateral discectomy was performed using the UBE technique (Heo et al., 2020). In 2022, a retrospective comparative study between the UBE technique and percutaneous endoscopic lumbar discectomy was conducted by a scholar (Jiang et al., 2022), which found that the two techniques had similar clinical efficacy. However, the UBE technique has more adequate exposure of the field, higher surgical safety, easier surgical operation, shorter operation time, and less radiation exposure of the operator.

UBE, a minimally invasive technique, offers several advantages, which mainly include: (1) the ability to achieve spinal canal decompression comparable to open surgery (Kim et al., 2018). (2) UBE causes less damage to the posterior spinal structures and can maintain the stability of the spine to a greater extent, without interbody fusion and internal fixation, and making postoperative recovery easier and hospitalization time shorter (Ito et al., 2021). (3) Continuous infusion of normal saline during UBE can keep the surgical field clear, remove inflammatory substances in time, and reduce the incidence of wound infection. At the same time, water pressure exerted pressure on venous vessels to reduce intraoperative blood loss. (4) The learning curve of UBE is relatively gentle, which is easy for spinal surgeons to master.

As a gradually emerging new technique, UBE has shown advantages in many aspects, but it still has certain complications. One study reported that the overall complication rate of UBE is about 10.3% (Choi et al., 2016), and its complications mainly include: dural tear, nerve root injury, spinal epidural hematoma, recurrence, inadequate decompression, and medical instability. Lewandrowski (Lewandrowski et al., 2021) et al. conducted a retrospective survey study of 93 endoscopic spine surgeons in 21 countries. The study counted 64,470 lumbar endoscopic procedures performed by these surgeons, of which 689 resulted in dural tears, and showed a 1.07% incidence of dural tears in lumbar endoscopic procedures. Most dural tears are small and can be successfully managed with mechanical compression with gelatin foam and sealants and 24–48 h of postoperative bed rest. 2/3 of patients with incidental dural tears have a completely uneventful postoperative recovery process. The remaining 1/3 of patients may have persistent cerebrospinal fluid leakage, nerve root injury with dysesthesia, sensory loss, or loss of motor function. In patients with lumbar spinal stenosis, the space between the dural sac and the vertebral plate narrows, and adhesions between the dural sac and the ligamentum flavum occur, leading to blind spots during unilateral approach bilateral spinal decompression surgery, resulting in dural tears. In addition, the ligamentous structure between the dural sac and the wall of the surrounding spinal canal, the spinal ligament, is closely connected to the ligamentum flavum. Thus this physiologic relationship, when the ligamentum flavum is pulled sharply, may result in part of the dural dorsal capsule being torn off along with the small blood vessels (Uchikado et al., 2020). Small folds may form on the surface of the dural sac under the combined effect of the spinal ligaments and saline pressure. When the spinal plate is removed using a Kirschner perforator, this may result in tearing of the dural sac together (Kim et al., 2020). In addition to the above, cerebrospinal fluid leaks, pseudo dural bulges, central nervous system infections, and surgical wound infections also occur.

Adequate documentation exists for recording diaphragmatic EMG using long-term placed metal electrodes in conducting experimental studies (Dow et al., 2006). Briefly, pairs of multi-stranded fine wire stainless steel electrodes were stripped to expose 0–3 mm conductive segments. A dissection is performed and the pair of electrodes, with the exposed portion of the wire implanted in the mid-rib region on either side of the diaphragm with an inter-electrode distance of 3 mm. the electrodes are tunneled and externalized on the back of the animal. The above modeling allowed real-time monitoring of changes in respiratory movements in rats.

We hypothesize that spinal cord-like hypertension is related to continuous intraoperative perfusion of water flow because UBE surgery is performed in a relatively confined spinal canal and must rely on continuous perfusion of saline to maintain a clear surgical field in order to perform the surgical operation smoothly. The epidural space being a continuous cavity, the magnitude of the perfused water pressure may have a functional effect on the cervical spinal cord or intracranial pressure. The cervical spinal cord or intracranial pressure is affected by changes in water pressure, resulting in respiratory function, but the exact extent of the effect on respiratory function is not well understood. In this study, we investigated the effects of different water pressures in the lumbar UBE on respiratory function.

## Results

### Changes in respiratory movement resulting from changes in perfusion pressure were associated with dural rupture

**FIGURE 1:**
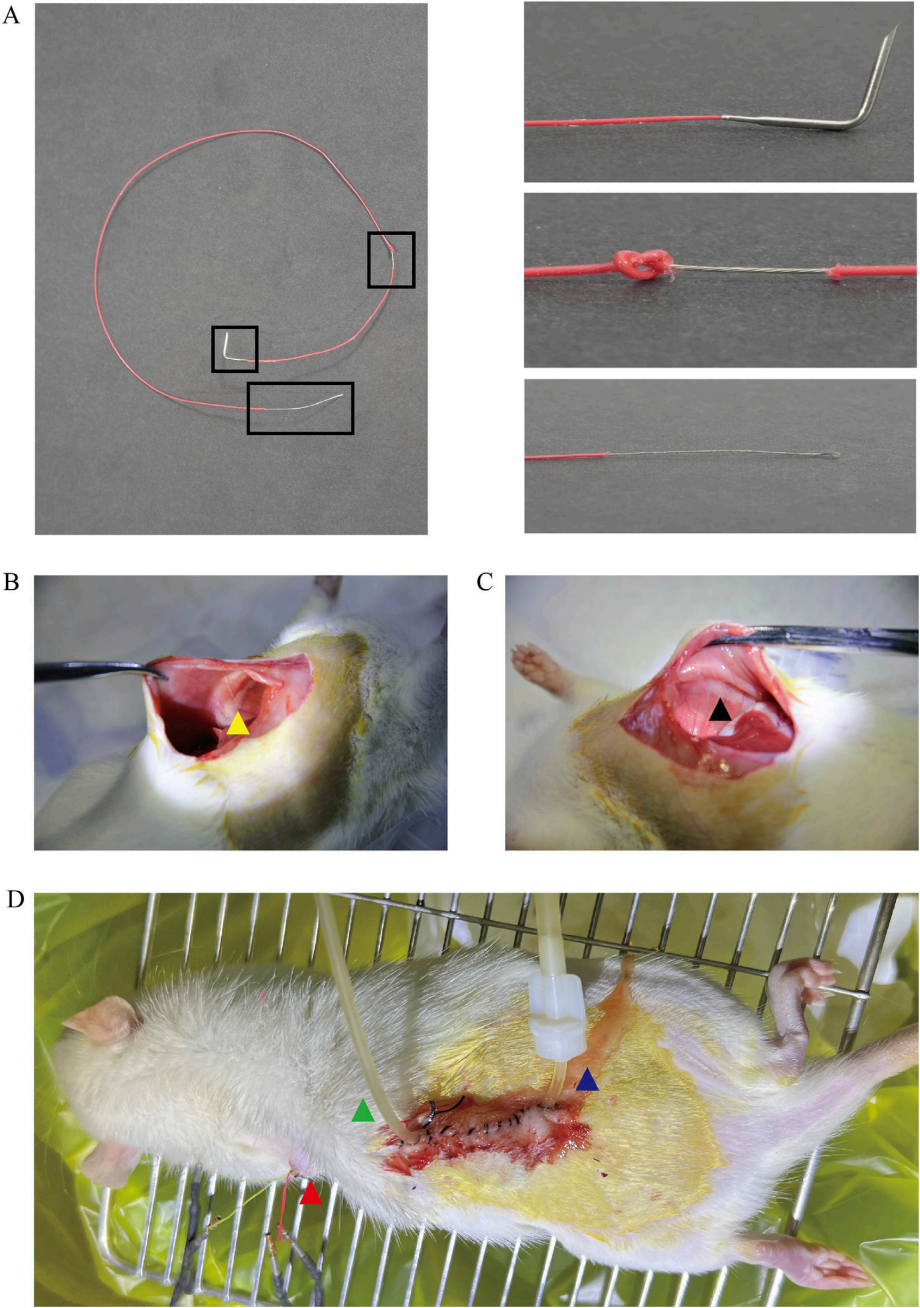
Schematic diagram of electrode and electrode implantation. **(A)** Pictures of electrodes: the three pictures on the right side show, from top to bottom, the needle end of the electrode, the part of the electrode in direct contact with the diaphragm, and the part of the electrode tail end connected to the signal receiver, respectively. **(B,C)** Yellow arrows show the implantation position of the right electrode, black arrows show the implantation position of the left electrode, and both implantation positions are at the junction of the diaphragm and white fat. **(D)** Cervical UBE surgical model: red arrow is the position of diaphragm electrode from the back, green arrow side of the hose connected to the pressure catheter, blue arrow direction catheter for saline perfusion channel.

**FIGURE 2:**
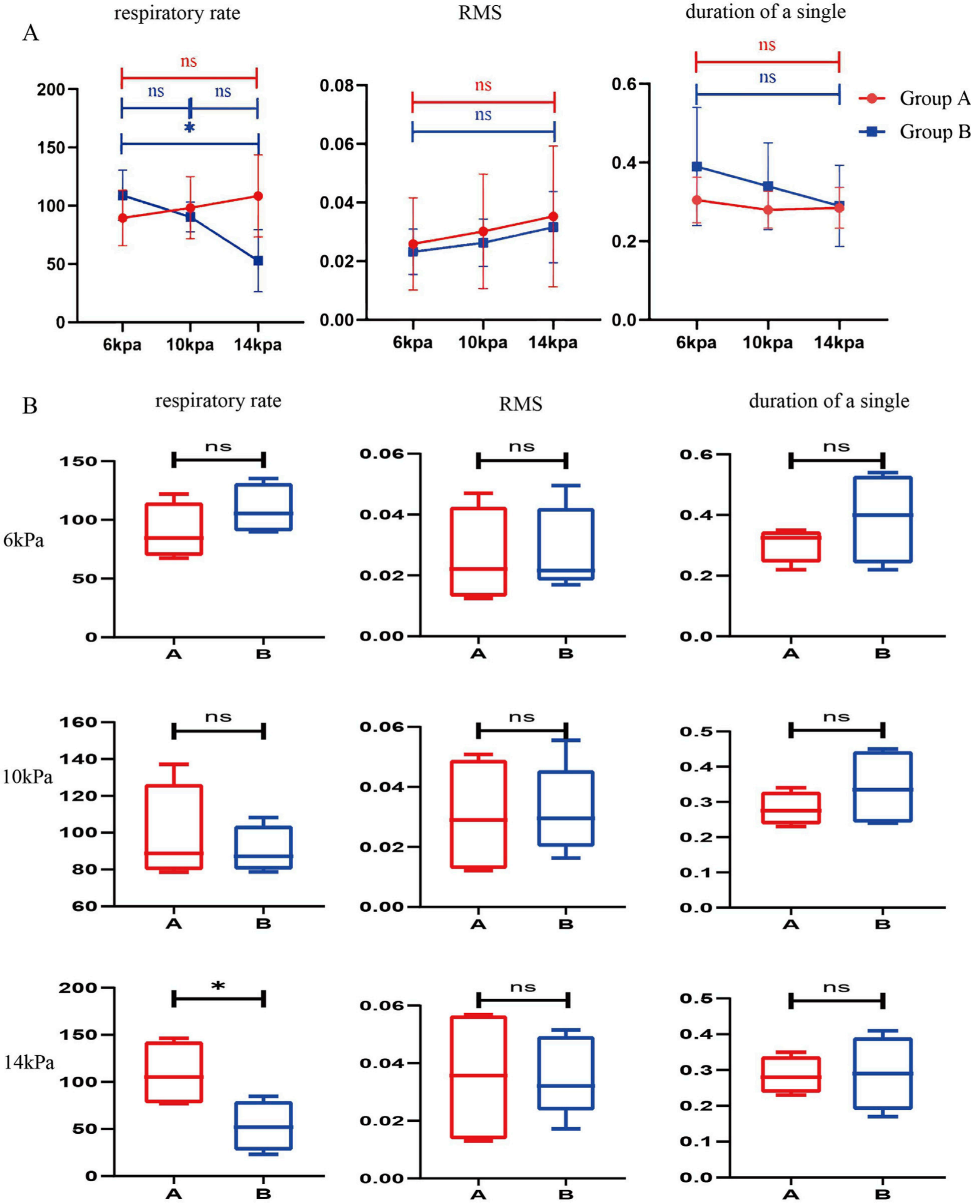
Comparison of respiratory data of rats under different perfusion pressures during the simulated surgical procedure. **(A)** Comparison of respiratory data of the same group of rats under different perfusion pressures. (n = 4) **(B)** Comparison of respiratory data of different groups of rats at the same perfusion pressure. (n = 4) All data are means ± SD, *P < 0.05.

To explore the effects of intraoperative dural injury and changes in perfusion pressure on respiratory motor function in rats (Figure 1). We simulated the UBE surgical procedure after modeling rats in both groups. The diaphragm electromyography signals of the rats were observed and recorded under the condition that the initial implanted electrodes were not perfused, and the perfusion pressure was maintained at 6 kPa, 10 kPa, and 14 kPa for 2 min, respectively. The resulting diaphragmatic electromyographic signals were organized and analyzed (Figure 2A).

The mean respiratory rate of four rats in group A was (89.59 ± 23.83) bpm at 6 kPa, (98.32 ± 26.59) bpm at 10 kPa, and (108.49 ± 35.31) bpm at 14 kPa, with P = 0.1560 > 0.05, which showed that the respiratory rate of the rats was not related to the changes in perfusion pressure. This data indicates that the respiratory rate of the rat is independent of changes in perfusion pressure when the dura mater is intact. The mean respiratory rate of the four rats in group B was (108.975 ± 21.536) bmp at 6KPa, (90.341 ± 12.761) bmp at 10KPa, and (52.903 ± 26.698) bmp at 14 KPa, P = 0.0130 < 0.05, which was statistically significant, and there was a significant difference. This data indicates that the respiratory rate of the rat decreases with increasing perfusion pressure during dural rupture. The RMS was (0.026 ± 0.016) at 6KPa, (0.030 ± 0.020) at 10KPa, (0.035 ± 0.024) at 14KPa, P = 0.2434 > 0.05, which showed that there was no significant correlation between the RMS and the changes in perfusion pressure. Single diaphragm movement time was (0.305 ± 0.058) seconds at 6KPa, (0.28 ± 0.047) seconds at 10KPa, (0.285 ± 0.052) seconds at 14KPa, P = 0.2823 > 0.05, there was no significant correlation between the time of single diaphragm movement and the change of perfusion pressure. There was no significant correlation between RMS, diaphragm single movement time and perfusion pressure changes (Figure 2B; Table 1).

**TABLE 1:** Detection of respiratory function in cohort rats during lumbar UBE surgery.

Observation indicators	Group	6 kpa	10 kpa	14 kpa	p value
Respiratory rate (bpm)	Group A	89.59 ± 23.83	98.32 ± 26.59	108.49 ± 35.31	0.1560
	Group B	108.975 ± 21.536	90.341 ± 12.761	52.903 ± 26.698	0.0130*
RMS	Group A	0.026 ± 0.016	0.030 ± 0.020	0.035 ± 0.024	0.2434
	Group B	0.023 ± 0.008	0.026 ± 0.008	0.032 ± 0.012	0.4833
Word breathing time (seconds)	Group A	0.305 ± 0.058	0.28 ± 0.047	0.285 ± 0.052	0.2823
	Group B	0.390 ± 0.15	0.340 ± 0.110	0.290 ± 0.103	0.5403

by one-way ANOVA, *p < 0.05.

### Rupture of the dura mater causes perfusion pressure to affect respiratory movement

**FIGURE 3:**
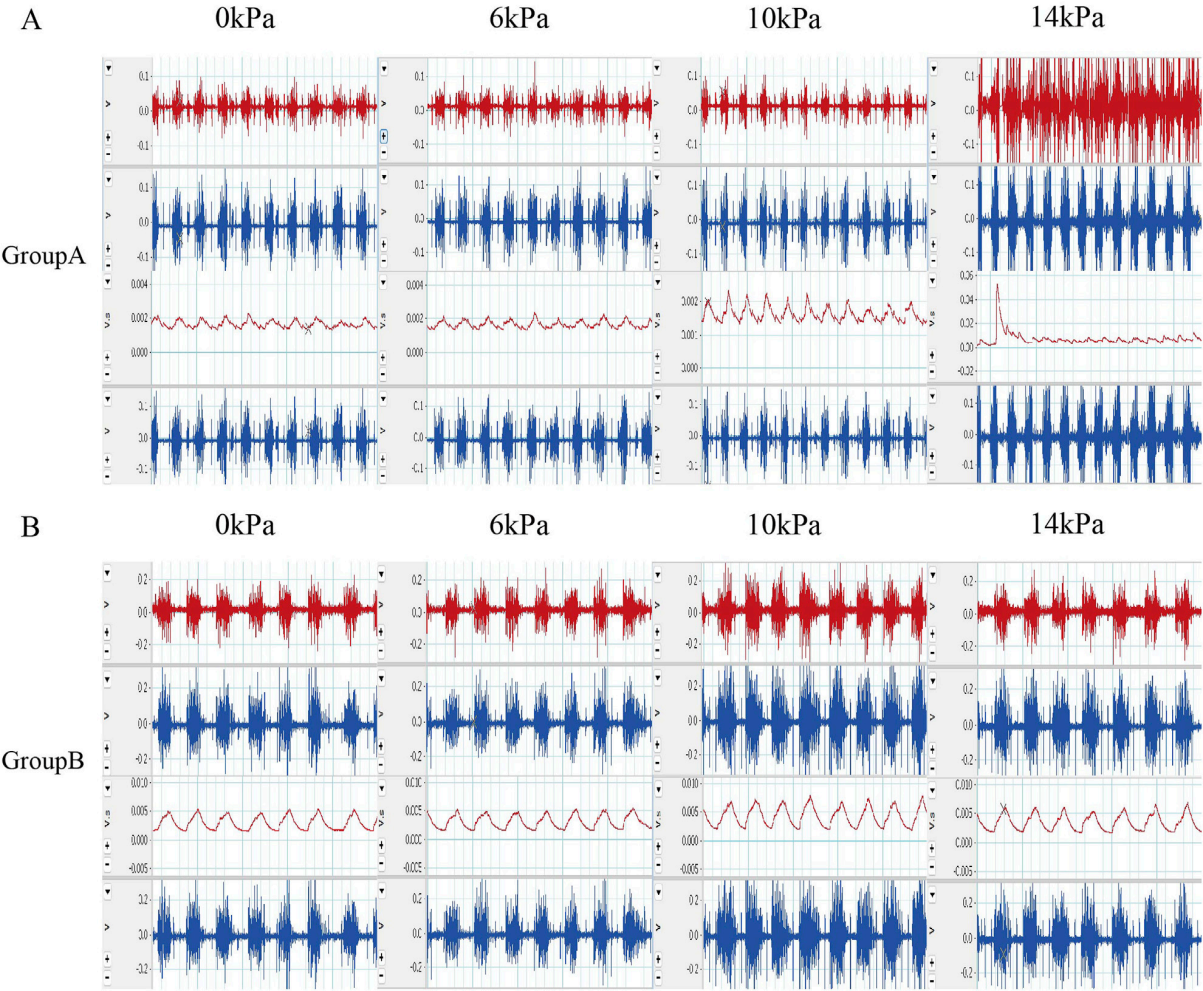
Original EMG and post integration EMG results **(A)** Raw EMG images and integrated EMG images of rats in group A during the experiment. **(B)** The EMG images of rats in group B during the experimental process and the EMG images after integration were selected.

To discuss further, the effect of perfusion pressure on respiratory movement during dural rupture, we analyzed the data obtained. The results showed that when the dura mater was ruptured, the increase of perfusion pressure led to a decrease in respiratory rate, but no significant changes in RMS and single respiratory rate were observed. The above results may indicate that changes in water pressure affect respiratory function by affecting respiratory rate after intraoperative dural rupture (Figure 3; Table 2).

**TABLE 2:** P-value for intergroup comparison of respiratory function data between rats in group A and group B at the same pressure.

Observation indicators	6 kpa	10 kpa	14 kpa
Respiratory rate (bpm)	0.2728	0.6078	0.0458*
RMS	0.7972	0.8699	0.9817
Word breathing time (seconds)	0.3321	0.3559	0.9339

by independent samples t-test, *p < 0.05

### The integrity of the spinal cord does not cause perfusion pressure to have a direct effect on the massive perfusion of the spinal cord

**FIGURE 4:**
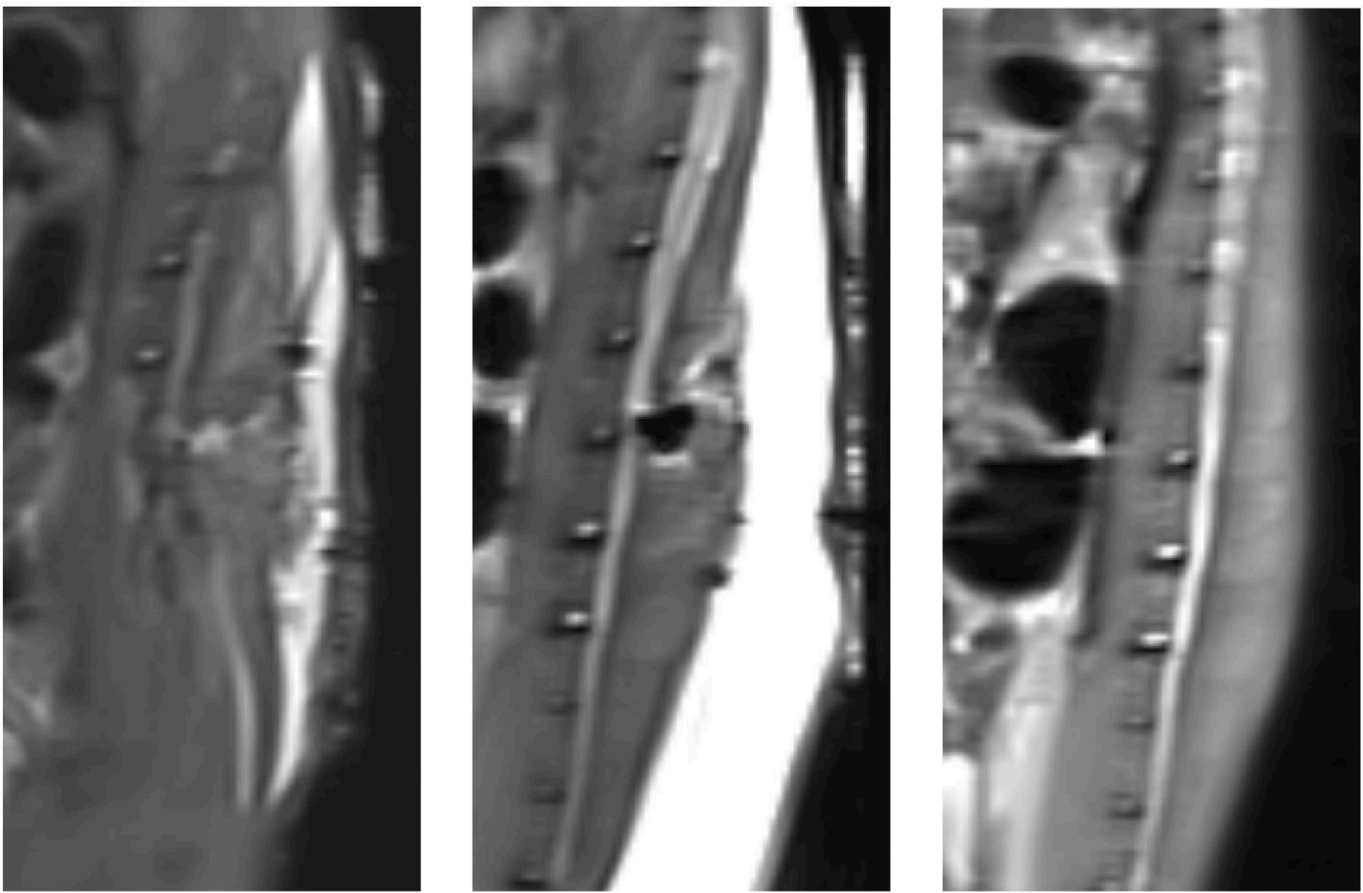
Magnetic resonance imaging results of postoperative rats and preoperative rats (T2): From left to right, the MRI results are shown for rats in group A, rats in group B, and rats that did not undergo surgery.

The results of MRI scans of the rat spinal cord after surgery and before surgery are shown in Figure 4. There was no obvious effusion in the spinal cord after operation. This indicated that there was no obvious fluid perfusion in the spinal cord when the dura mater was incomplete.

### Rupture of the spinal dura leads to cauda equina neurogenesis

**FIGURE 5:**
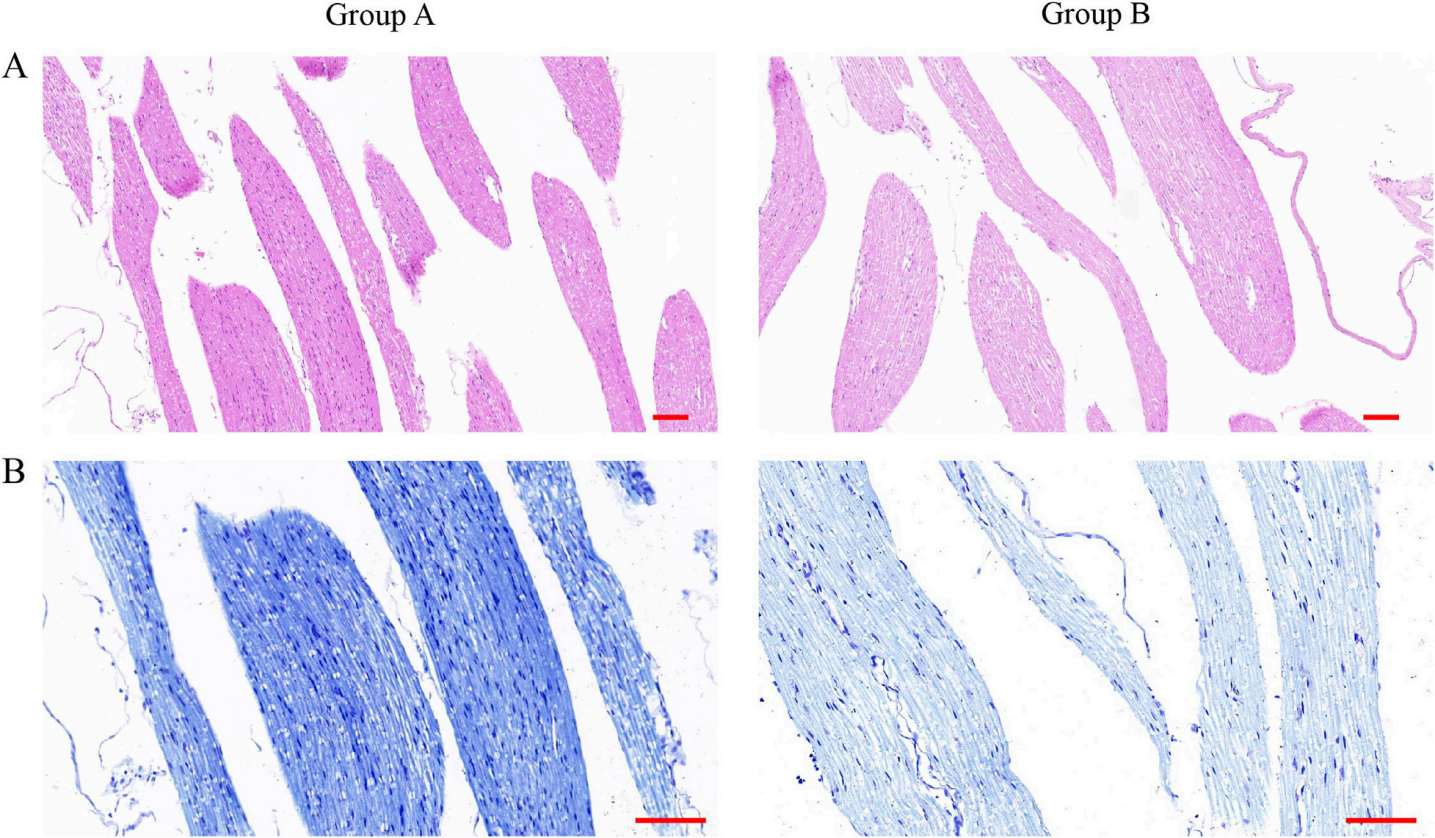
HE staining and toluidine blue staining of spinal cord slices from rats after surgery. **(A)** HE staining of spinal cord sections of rats in group A and group B. (Scale bar 100 μm). (Scale bar: 100 μm) **(B)** Toluidine blue staining of spinal cord sections from rats in group A and group B. (Scale bar, 100 μm).

**FIGURE 6:**
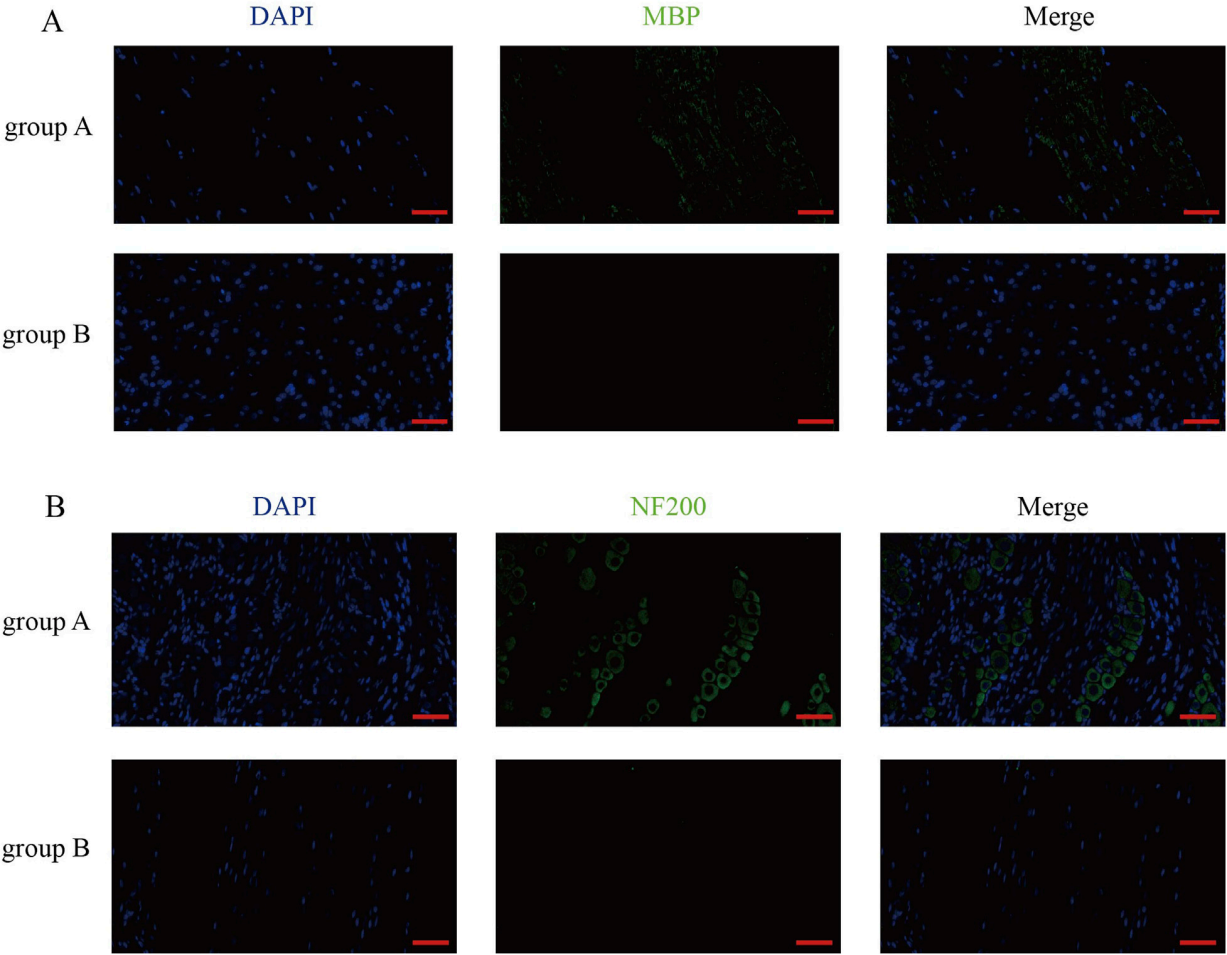
MBP staining and NF200 staining results of rat spinal cord sections after surgery. **(A)** MBP staining of rat spinal cord sections in group A and group B. (Scale bar, 50 μm). **(B)** NF200 staining of spinal cord sections from rats in group A and group B. (Scale bar, 50 μm).

In order to verify the effect of dura mater integrity on the nervous system, the cauda equina of rats was selected to make sections, and the pathological staining and immunofluorescence were performed. After counting Nissl body in HE-stained spinal cord sections, we found that there were more Nissl body in group B than in group A (Figure 5A). Similarly, the same results were obtained in toluidine blue-stained spinal cord sections (Figure 5B). By observing the results of sections stained with MBP and NF200, we found that the level of spinal cord injury in group B was higher than that in group A (Figures 6A,B).

### In the case of intraoperative perfusion, the dura mater can be seen as a protective device for the spinal cord

We can observe from the results that the change of respiratory rate and single breath time with the increase of water pressure in the dura mater rupture group is more obvious than that in the non-dura mater rupture group. The intraoperative perfusion had a greater effect on the vital signs of the rats after dural rupture. The effect was even smaller in rats with unruptured dura mater (Figure 2).

## Discussion

The establishment of an intravertebral perfusion system is necessary for UBE monitoring in rats. To ensure the accuracy and controllability of the intravertebral perfusion, we used real-time monitoring of the perfusion pressure. The pressure monitoring catheter was connected to the end of the perfusion tube to ensure that the whole perfusion channel was tightly connected to ensure the accuracy of pressure monitoring. Clinically, we usually use gravity perfusion, i.e., hanging the perfusion fluid to a certain height and relying on gravity for perfusion. In gravity perfusion, the perfusion speed and pressure will drop as the level of the perfusion fluid falls, so the perfusion speed and pressure are not constant. The pressure monitoring catheter can avoid the pressure difference caused by the drop of liquid level and the size of incision tension.

This experimental monitoring system mainly monitors the diaphragm electromyography of rats during the simulation experiment, and the electromyography can reflect the frequency of respiration, the relative magnitude of the amplitude of diaphragm movement, and the duration of a single diaphragm movement.

The implantation of diaphragm electrodes is one of the key steps. The Wistar rats used in this study were about 8 weeks old and weighed 240–300 g. The electrodes were implanted 1 day before the simulated operation, and the implantation positions were located at 3 and 9 points of the diaphragm. After the successful implantation of the diaphragm, the changes in respiratory rate and amplitude of thoracic movement of the rats were examined to determine the presence of concomitant pneumothorax. The electrode wire from the abdomen was passed through a tunnel from the subcutaneous to the back of the neck on the left side of the rat, and then from the dorsal skin to be fixed, in order to avoid the destruction of the electrode wire caused by the rat’s limb movement and biting, which would lead to the deviation of the experimental results. In some studies, they chose to implant electrodes in the ventral, medial, and dorsal regions of the diaphragm to measure diaphragm EMG signals to avoid the false-negative result that only one region of the diaphragm was detected and action potentials from undetected regions were lost (Kramer et al., 2014). However, this electrode implantation was ineffective and did not capture new diaphragm EMG signals. For example, in a study of 44 animals, some animals had no EMG activity in the middle portion of the diaphragm and no EMG activity in two other regions such as the ventral versus the dorsal portion (Warren et al., 2018). In addition, the thinness of the ventral and dorsal portions of the diaphragm is not conducive to suturing, and the dorsal portion is blocked by viscera, which increases the incidence of pneumothorax by forced suturing. Therefore, the implantation method as well as the implantation location selected in this study are desirable.

In this study, we used female rats to avoid rat death. As reported by M Farooque (Vinit et al., 2007), female mice on day 14 after thoracic 10 spinal cord impingement injury had less destruction of diseased spinal cord structures and higher BBB scores compared to male mice. This is similar to the clinical experience that female patients tend to achieve better clinical recovery after spinal cord injury than males (Golder and Mitchell, 2005; Golder et al., 1985).

In this experiment, rats were divided into two groups, the dura intact group and the dura rupture group, and this grouping was mainly related to the dural sac rupture, which is the most common complication in UBE surgery. Dural sac rupture is a common complication during endoscopic surgery when targeting dural sac decompression. Dural rupture is especially likely to occur in cases of severe stenosis of the degenerative spinal canal. When the dura is intact, the dura acts as a natural barrier to block the direct impact of the perfusate on the cauda equina. It also prevents the perfusate from entering the subarachnoid space. On the contrary, when the dura is broken, the perfusion can directly cause mechanical impact on the cauda equina, and the perfusate directly enters into the subarachnoid space, resulting in an increase in cerebrospinal fluid pressure (cranial pressure). Based on the above, the present study was designed for the intact dural group and the broken dural group.

In addition, we designed different perfusion pressures to observe the effects of different perfusion pressures on the respiratory motor function of rats. This is due to the fact that there is no standardized perfusion rate during clinical endoscopic surgery. Different doctors hang the perfusate at different heights. During intraoperative bleeding, it is necessary to increase the perfusion rate to maintain the surgical field in order to keep the field clear. However, in the case of prolonged perfusion at a higher perfusion rate, the patient may experience neck pain or other discomfort, leading to interruption of endoscopic surgery and surgical failure. The aim of this study is to theoretically solidify the effects of high-speed perfusion on the above clinical indicators from the aspect of basic experiments.

Intravertebral perfusion in the dura intact state does not lead to changes in respiratory function. In the present study, we found that the respiratory rate, RMS and single respiration time of rats in the dura intact state did not change much with pressure, regardless of low or high perfusion pressure. We believe that this experimental result is closely related to the perfusion rate, cranial and vertebral anatomy. Normally, the vast majority of the perfusate would outflow along the outlet of the working channel, but still a smaller amount of perfusate would diffuse cephalad and caudal in the relatively closed epidural space of the spinal canal (Vesovski et al., 2019; Hong et al., 2017). Since the spinal canal is interconnected internally and externally via the intervertebral foramina (Kinoshita et al., 2018; Jeong et al., 2017), the perfusate would diffuse bilaterally to the extracranial side of the spinal canal through multiple intervertebral foramina (Gill et al., 2020). This is consistent with our experimental results that none of the respiratory indexes of the rats in group A showed significant changes in the presence of elevated perfusion pressure.

The effect of perfusion in the dura-broken state on the respiratory function of rats was even greater. After the dura was broken during UBE, the perfusate would continue to enter into the subarachnoid space along the breach. The present study showed that the respiratory rate of rats decreased with increasing perfusion pressure (P < 0.05). We believe that the Cushing response induced by continuous perfusion in the dural rupture state (Barbiro-Michaely and Mayevsky, 2003; Stepińska et al., 1995; Pásztor and Pásztor, 1980; Barry et al., 2012) can better explain this change. The Cushing reflex is a physiologic neurological response of the nervous system to an acute increase in intracranial pressure, including the Cushing’s triad that appears as a widening of pulse pressure (increase in systolic blood pressure, decrease in diastolic blood pressure) bradycardia, and respiratory irregularities. During the first phase of the Cushing reflex, blood pressure and heart rate are elevated by sympathetic activation to overcome the increase in intracranial pressure. In the second phase of the Cushing reflex, hypertension continues, but the patient develops bradycardia rather than tachycardia. There is disagreement about the mechanism that leads to this phase of the Cushing reflex. The previous idea was that elevated blood pressure leads to activation of pressure receptors in the aortic arch, triggering parasympathetic activation and leading to bradycardia. It has since been proposed that extracerebral chemoreceptors are not involved in this phase and that bradycardia is actually caused by intracranial vagal compression. The perfusate diffuses in the subarachnoid space in addition to the spinal canal, and the Cushing reaction leads to a reduction in respiratory rate. There is still not complete agreement as to what causes bradycardia, but it is now widely recognized as a late and possibly terminal sign of worsening intracranial lesions. In the later stages of the Cushing reflex, brainstem dysfunction secondary to increased intracranial pressure, tachycardia, or bradycardia can be clinically manifested as irregular respiration; it is initially characterized by shallow breaths and occasional apneas. This activity occurs as a result of compression of the brainstem by increased intracranial pressure. Eventually, with the onset of brain herniation, dyspnea may develop and progress to respiratory and cardiac arrest. However, in the present experiment, the rats did not die at the end of the experiment, which may be related to the short duration of our continuous perfusion (we designed a continuous perfusion of about 10 min). Theoretically, the longer the duration of subarachnoid perfusion, the more likely it is to induce a severe Cushing response. Further experiments are needed to verify whether prolonged perfusion leads to severe Cushing reaction in rats.

In addition to the Cushing reaction mentioned above, “spinal cord like hypertension” is also used to explain this change in China. There are many symptoms of spinal cord hypertension, such as: dizziness, nausea, headache, chest tightness, shortness of breath, dyspnea, and apnea in severe cases (Joh et al., 2009). But at present, the pathogenesis of spinal cord hypertension syndrome is not clear, and many articles are discussing the possibility of spinal cord hypertension. During Unilateral Biportal Lumbar Endoscopic procedures, normal saline is usually used as a medium for flushing the channel, and it is necessary to use normal saline for continuous perfusion during the procedure. This is the reason why the rats were perfused with normal saline in our experiments. When the perfusion pressure of normal saline is increased or the pressure in the spinal canal is too large due to long-term perfusion, the pressure in the environment of the spinal cord microvessels is increased, and the microcirculation is blocked (Pásztor and Pásztor, 1980; Hurst et al., 1995). On the one hand, it can play a role in stopping bleeding during operation, on the other hand, it can also cause reflux obstruction. The result of the above situation is ischemia and hypoxia in the microenvironment of the spinal cord and nerve edema, and eventually there are many neurological symptoms, and respiratory changes are one of the results. However, in this experiment, we performed simulation experiments after rats were anesthetized, and some related symptoms that need subjective expression may not be measured. We chose the indicator of respiration and converted it into measurable electromyography for detection. However, the specific principle needs further experimental verification.

For these reasons, effective measures should be taken to avoid dural tears during surgery. First, the causes of dural tear were analyzed, and one of the important factors was the unclear microscopic field of view, which led to accidental damage to the dura mater. Intraoperative hemorrhage in the operative field often leads to blurring of the surgical field and affects the accuracy of operation. Effective hemostasis is therefore essential. Intraoperative methods to control intravertebral hemorrhage include: applying radiofrequency electrode electrocoagulation to stop hemorrhage, maintaining the patient’s systolic blood pressure below 100 mmHg, sealing the bone trauma with bone wax in a timely manner, and ensuring that the irrigated saline continues to flow out smoothly, etc. Second, the ligamentum flavum in the middle of the dura mater and vertebral plate has the function of a protective barrier, which can be removed during the operation after completing the adequate decompression of the bony structures, and in the meantime, it has indirect protective effect on the deeper neural structures. The ligamentum flavum can be removed after sufficient decompression of the bony structures has been completed, during which time it has an indirect protective effect on the deep neural structures. If the ligamentum flavum has serious adhesion with the dural sac, the ligamentum flavum should be peeled off carefully and gently, with moderate decompression, avoiding rough tearing and thus tearing the dura mater, and if the ligamentum flavum can be completely free and floating in the dura mater, the decompression effect has been achieved; and there are also spinal ligaments in a net-like structure, which are distributed in the dorsal dura mater and its vicinity (Shi et al., 2012), which are in the dorsal dura mater. This ligament plays a connecting role between the dorsal dura mater, the vertebral plate, and the ligamentum flavum, and should be emphasized. It has been suggested that incomplete intraoperative separation and resection of the spinal ligament is the main cause of accidental dural tears (Lee et al., 2021).

The shortcomings of this experimental study are in the inability of rats to provide the same informative feedback after UBE surgery as in humans. In addition, the spinal cord anatomy of rats differs from that of humans, with the presence of 6 lumbar and 13 thoracic vertebrae in rats, resulting in the surgical site being farther away from the cervical diaphragmatic motor neurons and respiratory centers, which, therefore, may affect the accuracy of the results of the study. In the actual clinical operation, compared with the intervertebral foramenoscopy technique, the UBE technique caused a larger amount of bone defect, and the treatment of the ligamentum flavum through the intervertebral plate approach also increased the risk of dural injury. The UBE technique is a general anesthesia operation, and the patient’s experience of the operation is better than that of the intervertebral foramenoscopy technique, but because of the failure to get timely feedback from the patient during the operation, there is a relatively higher risk of nerve root stimulation or injury; and the cost of general anesthesia is high, and the need for radiofrequency during the operation is high. The cost of general anesthesia is high, and the need for auxiliary equipment such as radiofrequency, plasma cutter head and grinding drill will increase the cost of surgery to a certain extent. Therefore, for patients with lumbar spine-related diseases who need surgery, it is necessary to consider various factors to select the appropriate surgical method, and a multicenter prospective randomized study with a large sample and long-term follow-up can be conducted to investigate the long-term efficacy of the UBE technique. In addition, the general anesthesia administered to the experimental rats is susceptible to over-anesthesia and even intraoperative death because of the lack of real-time monitoring of the rats’ oxygen saturation, body temperature, and pulse rate. Different depths of anesthesia in rats have different effects on the basal respiratory rate of rats. When the anesthesia is too deep, the rats will die during the operation. At the same time, there was a certain deviation in the results when comparing the experimental data between groups of rats, which will be further improved in future studies.

Although there are still some shortcomings in UBE surgery, with the popularization of UBE technology, more and more studies have confirmed its clinical advantages in the treatment of spine-related diseases. The UBE technique preserves more bone than traditional surgery, and soft tissue damage such as muscle ligaments around the spine is relatively reduced, maintaining spinal stability. Its surgical tools and endoscopes are not limited by a common working entrance, which greatly reduces the technical difficulty. Intraoperatively, the contralateral side reveals a good field of view, which facilitates contralateral decompression, while the use of common arthroscopic and spinal instrumentation helps to reduce medical costs. In addition, during operation, the operator can apply familiar conventional surgical instruments, and the UBE intraoperative field of view is similar to that of conventional surgery and microscopic surgery, so that the operator can adapt to the microscopic operation faster, which shortens the learning curve and is easy to promote. It is believed that UBE surgery will play a more important role in future clinical work.

## Conclusion

By comparing the respiratory function of the same group of rats under different perfusion pressures, and comparing the respiratory function of different rats under the same perfusion pressure, it was found that the increase of perfusion pressure had no significant effect on the respiratory rate of the rats without dura mater rupture, while the respiratory rate of the rats with dura mater rupture increased and decreased with the increase of pressure. The respiratory rate of rats with ruptured dura mater was lower than that of rats with unruptured dura mater at the same perfusion pressure. In conclusion, in the case of dural rupture during lumbar UBE surgery in rats, the perfusion water pressure has an effect on the respiratory rate of rats, higher water pressure will inhibit the respiration of rats, and the respiratory movement changes of rats after dural rupture are more sensitive to the changes of perfusion water pressure.

## Materials and methods

### Selection of experimental animals

Eight female Wistar rats were used in this study. The rats were purchased from SPF (Beijing) Biotechnology Co. Ltd. with an initial weight of 240–300 g. They were acclimatized to the environment for 3 days after arrival at the animal house. The rats were housed under a 12-h/12-h light-dark cycle, room temperature of 20°C ± 3°C, and humidity of 50% ± 20%, and were supplied with adequate specialized feed and clean drinking water. All animal experiment procedures were in accordance with protocols approved by Institutional Animal Care and Use Committee of Wuhan University.

### Preparation of perfusion tubes for simulating UBE surgery

The use of ordinary intravenous infusion pipeline cut off the front end of the infusion needle part, the tail is connected to the specification of 3,000 mL of 0.9% sodium chloride solution, about 4–6 cm from the end of the head of the infusion device with scissors to cut out a number of small holes in the infusion tube, to avoid cutting the infusion tube, to ensure that saline can be flowed out of the cut out of small holes, so as to achieve the effect of the simulation of the perfusion channel in the operation.

### Preparation of electromyography electrodes

Take the empty needle of the medical syringe, use scissors to intercept the 0.5 cm anterior end, pay attention to keep the cylindrical structure of the syringe, and keep the needle for spare use.

Red and yellow Teflon wires of 13 cm length were taken as electrode wires. Two single knots were tied at 3 cm from the head end, the two single knots were overlapped together, and the rubber skin was removed from the 1 cm outer edge of the trailing end of the electrode wire with a No. 15 small round surgical blade, and the rubber skin was removed from the outer edge of the wire by 3 mm in the direction of the head end of the needle close to the knotted point, exposing the metal core of the electrode wire.

Insert the tip end of the electrode wire into the tail end of the prefabricated needle, the depth of insertion is about 2mm–4mm, and then use the medical needle holder to clamp the tail end of the needle closed, so that the electrode lead wire core and the needle can be firmly fixed together.

At about 3 mm–4 mm from the end of the needle, the needle was bent in the direction of the needle opening with a medical needle holder, so that the direction of the needle opening was toward the concave side, and the needle was bent at an angle of 120°.

The final electrode was shown in Figure 1A.

### Preoperative preparation and anesthesia of rats

#### Preoperative preparation

(1) Dietary control: 24 h before the surgery, stop providing solid food to the rats, and only provide sufficient clean drinking water to reduce the risk of vomiting during the surgery, and also reduce the interference of digestive tract contents on the surgery.
(2) Environmental preparation: Make the surgical area clean and hygienic, prepare all surgical tools and equipment, and autoclave surgical tools in advance to reduce the chance of postoperative infection. In addition, make sure that the height and temperature of the operating table are suitable for the size and physiological needs of the rats.

### Implantation of diaphragm electrodes

Anesthesia was administered with 1%–4% isoflurane.

The electrode implantation method is the same as in previous literature (Soliman, 2015; Soliman, 2016)

(1) The anesthetized rats were placed in the supine position on the heating pad of the surgical operating table to help maintain a relatively constant body temperature, the skin of the abdomen and the back of the left upper limb of the rats was prepared by applying an electric hair straightener, and a 20 mL medical syringe was padded on the thoracic dorsum of the rats to facilitate the exposure of the diaphragm. A sterile cavity towel was spread after disinfecting the operation area twice with iodophor gauze.
(2) Firstly, the abdominal skin of the rats was cut with a scalpel, the abdominal skin was cut along the midline of the anterior abdominal wall with tissue scissors, the length of the incision was about 6 cm, the subcutaneous tissue was separated with scissors, and the abdominal white line was found. Lift the abdominal wall muscles, open the peritoneum by extending the abdominal white line, and extend it upward to the subxiphoid process and downward to the skin opening.
(3) Use saline-soaked sterile gauze to stuff to the top of the liver, so that the diaphragm is separated from the abdominal organs, to fully expose the diaphragm, if there are ligaments in the liver that can't be separated from the diaphragm, the ligaments can be carefully cut to separate the diaphragm, and the movements need to be gentle in order to avoid damage to the diaphragm.
(4) The bilateral electrode implantation points were selected at the edge of the diaphragm bilaterally, and the implantation positions were located at 3 and 9o’clock in the direction of the diaphragm, taking care to avoid the diaphragm blood vessels. The direction of needle insertion was perpendicular to the direction of the diaphragm muscle fibers. After insertion of the needle, the needle was pulled until the exposed metal segment of the electrode wire was tightly attached to the diaphragm or penetrated into the diaphragm, and a single knot was tied at the exit point to fix the electrode wire to avoid pneumothorax when sewing, and the needle was cut off, and the insulating skin on the outer layer of the wire was stretched to keep the metal core wire from being exposed to avoid contacting the abdominal cavity organs and causing interference with the electrical signals. Two electrodes were implanted in the left and right sides, a total of 4 electrodes, adjacent electrodes should be kept parallel with a distance of 3 mm. different color electrode wires were used bilaterally to differentiate the left and right sides, and the changes in the respiratory rate and the amplitude of the thoracic movement of the rats were examined after the fixation was completed in order to determine the presence of a concomitant pneumothorax. The suture position is shown in (Figures 1B,C).
(5) The muscle layer of the abdominal wall was sutured, and the electrode wires were secured by passing a suture through a loop in a single knot of 4 strands of wire at the point where the wires exited the abdominal wall muscle. An epidural puncture trocar was used to expand a tunnel under the skin, connecting the subcutaneous area of the back of the neck and the subcutaneous area of the abdomen, and the 4 electrode wires were threaded out of the puncture trocar and fixed to the skin of the back to prevent rats from tearing and scratching the electrode wires. Finally, the abdominal surgical incision was sutured and antibiotics were injected intraperitoneally with levofloxacin hydrochloride injection (4.5 mg/100 mL) to prevent wound infection.

### Experimental grouping

The experimental rats were randomly divided into two groups, A and B. Group A was the group with unruptured dura and group B was the group with simple dural rupture. All rats underwent diaphragmatic electrode implantation on the previous day, and in group A, the perfusion catheter was placed on the lateral side of the dura after removing the L5 or L6 vertebral plate, and the perfusion pressure test was performed after tightly suturing the incision. in group B, the dura of the dorsal side of the L5 or L6 segments was cut after removing the L5 or L6 vertebral plate, and the perfusion catheter was placed on the surface of the spinal cord, and the perfusion pressure test was performed after tightly suturing the incision.

#### Molding operation

##### Group A laminectomy

(1) The anesthetized animals were placed prone on the experimental animal operating table, and after fixing the limbs, the hairs around the middle of L1-S2 on the lumbar back of the rats were prepared with an electric hair clipper, and after disinfecting with iodine vapor for three consecutive times, the surgical area was routinely covered with sterile perforated towels, and the operator wore a disposable sterile cap, disposable medical masks, and surgical gloves in the process of the operation, and all the surgical instruments were sterilized, and the surgical process strictly followed the principle of asepsis. All surgical instruments were sterilized, and the surgical process strictly followed the principle of asepsis.
(2) Position the rat sacrum, initially locate the rat lumbar vertebrae using the rat spinous process upward, cut the skin with a scalpel, separate the subcutaneous tissues with tissue scissors, use a spacer to prop open the rat skin, locate the first lumbar vertebrae spinous process (the first lumbar vertebrae of the rat and the first silver-white tendon of the lumbar part of the rat meet), and reconfirm whether the location is accurate, and then use the scalpel to locate accurately the first lumbar vertebra along the spinous process of the rat spine from the L4 vertebral body to the caudal side. After accurate positioning, a 3-cm-long incision was made along the rat spine from the L4 vertebral body to the caudal side with a scalpel to incise the fascia, bluntly detach the paravertebral muscles, expose the vertebral body and then use a retractor to support the incision, fully revealing the spinous processes and vertebral plates of the L4-L6 segments.
(3) Bluntly peel off the paravertebral muscles and the soft tissues attached to the vertebral plate of the L5-L6 segments, and expose the L5-L6 vertebral plate exactly.
(4) Lift the spinous process of L5 vertebra slightly upward with the forceps in the left hand to make the spine bowed, and destroy the L5-L6 articular synchondrosis with the bone-biting forceps in the right hand, paying attention to avoiding damage to the spinal cord and dura mater; use the bone-biting forceps to bite off some of the vertebral plates on one side, paying attention to avoiding intervertebral venous plexus hemorrhage and spinal cord injury, and the ligamentum flavum will be exposed after the removal of the vertebral plates, and then the ligamentum flavum is removed, taking care of keeping the dura mater intact to reconfirm that there is no obvious neurovascular injury and spinal cord injury. The dura mater was removed, and the dura mater was kept intact.

#### Establishment of dural injury model in group B

(1) Place the anesthetized animals prone on the experimental animal operating table, fix the limbs and then use an electric hair dresser to prepare the hair around the middle of L1-S2 on the lumbar back, and after disinfecting with iodine volts for three consecutive times, routinely cover the surgical area with a sterile perforated towel, and the operator wears a disposable sterile cap, a disposable medical mask, and surgical gloves in the process of the operation, and all the surgical instruments are sterilized, and the surgical process is strictly in accordance with the principle of sterility. The principle of asepsis was strictly followed in the surgical process.
(2) Position the rat sacrum, initially locate the rat lumbar vertebrae using the rat spinous process upward, cut the skin with a scalpel, separate the subcutaneous tissues with tissue scissors, use a spacer to prop open the rat skin, locate the rat first lumbar vertebrae spinous process (the first lumbar vertebrae of the rat and the first silver-white tendon membrane of the lumbar part of the rat meet), and reconfirm whether the location is accurate, and then use the scalpel to locate accurately along the rat spine spinous process from the L4 vertebrae to the caudal side on either side of the rat. After accurate positioning, a 3-cm-long incision was made along the rat spine from the L4 vertebral body to the caudal side with a scalpel to incise the fascia, bluntly detach the paravertebral muscles, expose the vertebral body, and then use a retractor to support the incision, to fully reveal the spinous processes and vertebral plates of the L4-L6 segments.
(3) Bluntly peel off the paravertebral muscles and the soft tissues attached to the vertebral plate of the L5-L6 segments, and expose the L5-L6 vertebral plate exactly.
(4) Lift the spinous process of L5 vertebra slightly upward with the forceps in the left hand to make the spine bowed, and destroy the L5-L6 articular synchondrosis with the bone-biting forceps in the right hand, paying attention to avoiding damage to the spinal cord and dura mater; use the bone-biting forceps to bite off some of the vertebral plates on one side, paying attention to avoiding intervertebral venous plexus hemorrhage and spinal cord injury, and the ligamentum flavum will be exposed after the removal of the vertebral plates, and then the ligamentum flavum is removed, taking care of keeping the dura mater intact to reconfirm that there is no obvious neurovascular injury and spinal cord injury. Remove the ligamentum flavum, keep the dura mater intact, and reconfirm that there is no obvious neurovascular or spinal cord injury.
(5) After removing the ligamentum flavum from L5-L6, use small toothed forceps to hold the dura mater of L5 segment, lift up the dura mater, and then use the scalpel blade to pick through the dura mater with a reversed direction. At this time, the cerebrospinal fluid will flow out instantly, and the blood stains on the surface of the dura mater will disappear instantly with the naked eye, and the incised dura mater will expand the incision due to the contraction of the cut dura mater. A cotton swab is dipped into the outflow of cerebrospinal fluid.

### Simulation of UBE surgical modeling

#### Group A rat model establishment

(1) The anesthetized animals were placed prone on the experimental animal operating table, after fixing the limbs, the hair in the area around the middle of L1-S2 on the lumbar back was prepared with an electric hair dresser, after disinfecting with iodine volts for three consecutive times, the surgical area was routinely covered with a sterile perforated towel, and the operator wore a disposable sterile cap, a disposable medical mask, and surgical gloves during the operation, and all the surgical instruments were sterilized, and the surgical process was strictly in accordance with the principle of asepsis was strictly followed in the surgical process.
(2) Position the rat sacrum, initially locate the rat lumbar vertebrae using the rat spinous process upward, cut the skin with a scalpel, separate the subcutaneous tissues with tissue scissors, use a spacer to prop open the rat skin, locate the rat first lumbar vertebrae spinous process (the first lumbar vertebrae of the rat and the first silver-white tendon membrane of the lumbar part of the rat meet), and reconfirm whether the location is accurate, and then use the scalpel to locate accurately along the spinous process of the rat vertebrae by the L4 vertebral body on both sides to the caudal side. After accurate positioning, a 3-cm-long incision was made along the rat spine from the L4 vertebral body to the caudal side with a scalpel to incise the fascia, bluntly detach the paravertebral muscles, expose the vertebral body, and then use a retractor to support the incision, to fully reveal the spinous processes and vertebral plates of the L4-L6 segments.
(3) Bluntly peel off the paravertebral muscles and the soft tissues attached to the vertebral plate of the L5-L6 segments, and expose the L5-L6 vertebral plate exactly.
(4) Lift the spinous process of L5 vertebra slightly upward with the forceps in the left hand to make the spine bowed, and destroy the L5-L6 articular synchondrosis with the bone-biting forceps in the right hand, paying attention to avoiding damage to the spinal cord and dura mater; use the bone-biting forceps to bite off some of the vertebral plates on one side, paying attention to avoiding intervertebral venous plexus hemorrhage and spinal cord injury, and the ligamentum flavum will be exposed after the removal of the vertebral plates, and then the ligamentum flavum is removed, taking care of keeping the dura mater intact to reconfirm that there is no obvious neurovascular injury and spinal cord injury. and spinal cord injury.
(5) The perforated part of the pre-made UBE surgical perfusion tube was placed on the surface of the spinal cord, with the direction of the tube parallel to the long axis of the rat’s torso and the head end facing the side of the head. The cephalic side was left about 3 cm of the tubing on the outer side of the epidermis.
(6) The incision on the back of the rat was tightly sutured layer by layer, and the two ends of the tubing were sutured and fixed at the site where the tubing penetrated the skin.
(7) The head end of the tubing was connected to a pressure detection catheter, and the tail end was connected to 3L of saline to ensure the integrity and tightness of the tubing connection.
(8) Connect the diaphragm electromyography measuring instrument, and turn off the unnecessary electronic devices around to exclude electromagnetic signal interference.
(9) Before the simulated UBE surgery experiment began, the rats were placed on the hollow partition below to avoid the saline oozing from the incision entering the respiratory tract of the rats and causing respiratory dysfunction.
(10) To start the experiment, open the flow valve on the infusion tube, adjust the flow rate to the maximum, and wait until the respiratory movement of the rats is stabilized to carry out the next step of pressure-raising experiments, and the starting perfusion pressure is 6 KPa.
(11) Group A and B experiments continue to raise the saline height, saline height can lead to increasing perfusion pressure, to the perfusion pressure of 10KPa, 14 KPa when the suspension of pressure, wait for 2 min on the computer to mark the current pressure value, and then continue to raise the pressure.
(12) After recording, close the flow valve, remove the perfusion tubing, suture the incision, and observe the rats until they awaken.

#### Group B rat model establishment

(1) Place the anesthetized animals prone on the experimental animal table, fix the limbs and then use an electric hair dresser to prepare the hair in the area around the middle of the L1-S2 on the lumbar back, and after disinfecting with iodine volts for three consecutive times, routinely cover the surgical area with a sterile perforated towel, and the operator wears a disposable sterile cap, a disposable medical mask, and surgical gloves in the operation process, and all the surgical instruments are sterilized, and the surgical process is strictly in accordance with the aseptic principle.
(2) Position the sacrum of the rat, initially locate the lumbar vertebrae of the rat by using the rat spinous process upward, cut the skin with a scalpel, separate the subcutaneous tissues with tissue scissors, and use a spacer to open the skin of the rat and locate the first lumbar vertebrae of the rat with the rat spinous process in the position as described above, and then reconfirm whether the positioning is accurate, and then use a scalpel to make an incision of about 3 cm along the spinous process of the rat spine on both sides of the rat spine, and make a 3-cm incision along the caudal side of the rat spine, cut the fascia was bluntly separated from the paravertebral muscles, and the vertebral body was exposed to fully reveal the spinous processes and vertebral plates of the L4-L6 segments by using a retractor to open the incision.
(3) Bluntly detach the paravertebral muscles and the soft tissues attached to the vertebral plate of the L5-L6 segments, and expose the L5-L6 vertebral plate exactly.
(4) Lift the spinous process of L5 vertebra slightly upward with the forceps in the left hand to make the spine bowed, and destroy the L5-L6 articular synchondrosis with the bone-biting forceps in the right hand, paying attention to avoiding damage to the spinal cord and dura mater; use the bone-biting forceps to bite off some of the vertebral plates on one side, paying attention to avoiding intervertebral venous plexus hemorrhage and spinal cord injury, and the ligamentum flavum will be exposed after the removal of the vertebral plates, and then the ligamentum flavum is removed, taking care of keeping the dura mater intact to reconfirm that there is no obvious neurovascular injury and spinal cord injury. The ligamentum flavum is removed, and the dura mater is kept intact.
(5) After removing the ligamentum flavum from L5-L6, use small toothed forceps to hold the dura mater of L5 segment, lift up the dura mater, and then use a razor blade to break the dura mater with a reverse direction. At this time, the cerebrospinal fluid will flow out instantly, and the blood stains on the surface of the dura mater will disappear instantly with the naked eye, and the incised dura mater will expand the incision due to the contraction of the cut dura mater. Dip a cotton swab into the outflow of cerebrospinal fluid.
(6) The perforated part of the UBE surgical perfusion tubing made in advance was placed on the surface of the spinal cord, and the tubing was oriented parallel to the long axis of the rat’s torso, with the head end facing the cephalic side. The cephalic side was left about 3 cm of tubing on the lateral side of the epidermis.
(7) The incision on the back of the rat was tightly sutured layer by layer, and the two ends of the tubing were sutured and fixed at the area where the tubing penetrated the skin.
(8) The head end of the tubing was connected to a pressure detection catheter, and the tail end was connected to 3L of saline to ensure the integrity and tightness of the tubing connection.
(9) Connect the diaphragm electromyography measuring instrument, and turn off the unnecessary electronic equipment around to exclude electromagnetic signal interference.
(10) Before the simulated UBE surgery experiment began, the rats were placed on the hollow partition below to avoid the saline oozing from the incision entering the respiratory tract of the rats and causing respiratory dysfunction.
(11) To start the experiment, open the flow valve on the infusion tube, adjust the flow rate to the maximum, and wait until the respiratory movement of the rats is stabilized to carry out the next step of pressure-raising experiments, and the starting perfusion pressure is 6 KPa.
(12) Group A and B experiments constantly raise the height of saline, saline height can lead to increasing perfusion pressure, to the perfusion pressure of 10KPa, 14 KPa when the suspension of pressure, wait for 2 min on the computer to mark the current pressure value, and then continue to raise the pressure.
(13) After recording, close the flow valve, remove the perfusion tubing, suture the incision, and observe the rats until they wake up.

The above rat model was completed as shown in (Figure 1D)

### Detection and analysis of electromyographic signals

The electrode wires were connected to the terminal connector of the signal amplifier, which in turn was connected to the data acquisition device (PowerLab), with the electrode wires on the right side of the diaphragm connecting to channel 1, and the electrode wires on the left side connecting to channel 2, a connection that could facilitate subsequent spinal cord injury model preparation. The pressure monitoring catheter was connected to another data acquisition device. The two data acquisition devices were connected to a computer and used in tandem on the data analysis software (LabChart 8 Reader), which allowed simultaneous recording of data from both acquisition devices in the same interface. The magnification is 2000 times, and the bandpass filter is set to 20Hz-1 kHz to avoid interference from external noise, such as the shadowless lamp of the test bench and the power adapter of the computer, etc. The results can be analyzed by LabChart 8 Reader. Experimental results can be analyzed and recorded with LabChart 8 Reader software.

LabChart 8 can integrate the EMG signal to obtain the data of respiratory frequency: after obtaining the EMG data without interference, select Integral type in the Integral Calculation dialog box to calculate the absolute value of the original data (Absolute Value), and select Time in the reset type (reset type). Constant Decay in the reset type, the time is 0.1s, click OK to get the respiration curve. In the Data Pad, click on the channel where you have just performed the integration operation, and select Average Cyclic Rate as the data to be displayed under the Cyclic Measurements option in the dialog box. In the dialog box, under Cyclic Measurements, select Average Cyclic Rate as the data to be displayed. In the image, select a segment of data to be displayed and click Add to Data Pad to see the respiration rate of the rat during the selected period of time in beats/minute in the Data Pad.

The root mean square (RMS) of rat EMG and the duration of a single breath can also be calculated and measured directly by the software (Mantilla et al., 1985; Mantilla et al., 2011). RMS is the amplitude of a single breath of rat EMG, and the magnitude of RMS is positively correlated with the amplitude of diaphragm movement, which is an indication of tidal volume of rats. Root-mean-square (RMS) EMG amplitude was calculated using a 50-ms moving window, and the last three diaphragmatic movements maintained at different pressures were selected for measurement, excluding only occasional spontaneous deep breaths (defined as inspiratory bursts greater than twice the root-mean-square EMG amplitude of normal pressure).

EMG signals were shown in Figure 3.

### Postoperative MRI in rats

The rats, which had not been awakened after the operation, were fixed in the prone position on the small animal MRI coil, and the coil was fixed on the scanning table to scan the thoracolumbar segment of the spine to the inferior segment of the cauda equina, and anesthesia could be added before scanning to ensure the image effect if necessary; the parameters were as follows: Spin Echo,SE, TR 3800 ms, TE 62 ms, layer thickness 2 mm, number of layers 10, FOV 100 mm × 100 mm, matrix 320 × 320. The postoperative MRI of rats was observed using the KOSAI DICOM Viewer to assess the postoperative changes in the spinal canal.

### HE staining

#### Tissue perfusion sampling

The rats were put to death at the end of the operation, the abdominal and thoracic cavities were incised, the pericardium was cut open, the right auricle was cut open, and the left ventricle was punctured using a 50 mL syringe filled with 4% paraformaldehyde, and the solution was injected using a syringe, if crimson blood was seen flowing out of the right auricle then the puncture was successful, and a curved forceps was used to add a needle, which was fixed on the pericardium, and the syringe was pushed in slowly until the liver became grayish white, and the fluid that flowed out could be seen to be colorless and transparent, proving the success of the perfusion. After the fluid was colorless and transparent, muscle tonus of the rat could be observed, proving that the perfusion was successful. Cut down the target segmental spine, eliminate the paravertebral muscles, remove the vertebral plate with laminar scissors, take care not to damage the dura mater, pick up the spinal cord with a nerve stripper, and cut the nerve root with a razor blade. The dura mater was stripped after removal. The specimens were soaked into 15 mL EP tubes in 4% paraformaldehyde (4°C) for 1 day, and the EP tubes were labeled with the rat number, specimen name, and date of experiment. Store in a refrigerator at 4°C.

#### Tissue embedding

(1) ethanol dehydration: tissues were dehydrated by different concentrations of ethanol solution step by step 75% 85% 95% 100% 100%, all levels of ethanol for 1 h (Note: bony and calcified tissues need to be decalcified in decalcification solution decalcification solution, to be decalcified in the bone tissues and then carry out the step of ethanol dehydration);
(2) Transparency: the tissues were sequentially immersed in 2 xylenes, I cylinder each 20 min; II cylinder
(3) Wax immersion: the tissues were sequentially immersed in three paraffin wax cylinders, the first cylinder for 1 h, the second cylinder for 1.5 h, and the third cylinder for 2 h;
(4) Embedding: the liquid paraffin wax was poured into a mold box, and then the wax-impregnated tissues were placed on the bottom of the mold box flatly, with attention to the direction of the cut surface placed in the downward direction, and then the embedding box was removed when the paraffin wax was solidified, and then the wax block was trimmed after completely cooling down and becoming hard, and the paraffin wax in the periphery of the tissues was retained in order to be cut into slices. The periphery of the tissue is retained moderately for slicing.

#### Slicing

The pre-cooled wax block was fixed on the paraffin slicer so that the cut surface of the block was parallel to the knife, and the knife was usually inclined at 15°. The thickness of the slices was adjusted to 4 μm by turning the rotary advancer, and the slices were cut to a uniform thickness. The left hand holds the brush, the right hand rotates the slicer handle, after the slices are brought out, the brush is used to gently hold up the brush, and then tweezers are used to gently tweeze the wax slices, and then the slices are put into the spreading box in a positive way, and the water temperature is about 40°C. The slices are then put into the spreading box. Fish out the slices after spreading. Attach the slides with one end of the slide in the left hand, vertically into the water to attach the slices, the right hand with tweezers to assist in promoting the attachment to two-thirds of the slide. After attaching the slice, put it in the air to dry a little, put it on the 60°C baking machine for 1 h, and then put it in the oven for 1 h.

#### HE staining

A. Paraffin section dewaxing to water: sequentially put the paraffin section into xylene I for 20 min-xylene II for 20 min-xylene III for 15 min-anhydrous ethanol I for 5 min-anhydrous ethanol II for 5 min-85% alcohol for 5min-75% alcohol for 5 min-wash with distilled water. b. Nuclei staining: the sections were stained with Harris hematoxylin for 5–10 min, washed with tap water, and 1% of the nuclei were stained. c. Nuclei staining: the sections were stained with Harris hematoxylin for 5–10 min, washed with tap water, and then washed with 1% of the cellular material. The sections were then washed with tap water. c. Cytoplasmic staining: the sections were stained in eosin stain for 1–3 min d. Dewatering and transparent sealing: the sections were sequentially placed in 75% alcohol for 5 min - 85% alcohol for 5 min - 95% alcohol for 5 min - anhydrous ethanol I for 5 min - anhydrous ethanol I for 5 min e. Dewatering and transparent sealing: the sections were sequentially placed in 75% alcohol for 5 min - 85% alcohol for 5 min - 95% alcohol for 5 min - anhydrous ethanol I for 5 min - anhydrous ethanol I for 5 min. Anhydrous ethanol I 5min -Anhydrous ethanol II 5min -Xylene I 5min Dewatering transparent, neutral gum sealing.

Sections were scanned using a panoramic scanner to preserve the image of the anterior horn of the spinal cord, and the experimental results were observed using SlideViewer software.

### Toluidine blue staining

Tissue perfusion sampling, tissue embedding and sections were made in the same way as HE staining.

(1) Receive first the sectioned wax block for dehydration. The specimens are immersed in the following experimental reagents and for the appropriate time. Xylene I for 5 min, Xylene II for 15 min, Xylene III for 15 min, anhydrous ethanol for 1 min, 95% ethanol for 1 min. After immersion, rinse with purified water.
(2) Prepare the section after rinsing, take an appropriate amount of toluidine blue staining solution, flatten the section, and then add toluidine blue staining solution for staining, the staining time is about 20–30 min.
(3) When it is observed that the section has been completely stained, rinse it lightly with running water to remove the excess staining solution on the section.
(3) Use 95% ethanol for color separation and observe the color separation effect under the microscope.
(4) Finally, the slices were dehydrated and transparent, and were immersed in the following reagents for a corresponding period of time, anhydrous ethanol for 1 min, xylene 3 times for 1–2 min each time, and finally sealed with neutral adhesive.

### MBP staining and NF200 staining

Tissue perfusion sampling, tissue embedding and sections were made in the same way as HE staining.

(1) paraffin section dewaxing to water: firstly, put the paraffin section into xylene Ⅰ soaking for 20 min - xylene Ⅱ soaking for 20 min - xylene Ⅲ soaking for 20 min - anhydrous ethanol Ⅰ soaking for 3 min - 90% alcohol soaking for 3 min - 80% alcohol soaking for 3 min - 70% alcohol soaking for 3 min, take out the specimen, and then wash it with distilled water.
(2) antigen repair: the tissue sections were placed in the autoclave containing EDTA antigen repair buffer for repair (PH value control at 9.0), start the autoclave, jet timing for 3 min closely observe to prevent excessive evaporation of buffer caused by dry slides. At the end of the jet, the tissue sections were cooled naturally, and the slides were soaked and washed in PBS at pH = 7.4 for 3 min each time.
(3) Serum closure: After the sections were lightly shaken dry, use a histochemical pen to draw a circle around the tissue to prevent the antibody from flowing away, and add BSA at a concentration of 1% dropwise inside the drawn circle, so that it covers the tissue uniformly. In the room temperature environment, closed for 60 min.
(4) Add primary antibody: Shake off the sealing solution gently, add a drop of primary antibody in a certain proportion on the section, place the section horizontally in a wet box, add a small amount of water into the wet box to prevent the antibody from evaporating, and incubate at 4°C overnight.
(5) Add secondary antibody: take out the sections after primary antibody incubation, soak them in PH = 7.4 PBS for 3 times, each time for 3 min after a little shaking off, under the condition of avoiding the light, add the fluorescent secondary antibody of the corresponding genus to the primary antibody dropwise inside the drawn circle, cover the tissues, and then incubate the sections for 1 h under the condition of avoiding the light and at the temperature of room.
(6) DAPI re-staining of cell nuclei: take out the incubated sections, place them in PBS (PH7.4) again and wash them for 3 times, each time for 10 min after slight drying, add the appropriate amount of DAPI nuclei staining solution into the circle, and then stain them for 5 min at room temperature.
(7) Observation sealing: take out the stained section, put it in PBS (PH7.4), wash it fully 3 times, each time for 5 min under the fluorescence microscope, observe whether the target antibody and the cell nucleus fluorescence are stained successfully or not, and then seal the section with the anti-fluorescence quenching sealer.

### Statistical analysis

The experimental results were organized and analyzed using GraphPad Prism 8 software, and statistical graphs were drawn. One-way ANOVA was used to compare whether respiratory rate, peak RMS and single respiration time were related to changes in perfusion pressure within groups, and independent samples t-test was used to determine whether respiratory rate, peak RMS and single respiration time were related to changes in perfusion pressure between groups. p < 0.05 indicated statistical significance.

## Data Availability

The original contributions presented in the study are included in the article/supplementary material, further inquiries can be directed to the corresponding author.

## References

[B1] Akbary K. Kim J. S. Park C. W. Jun S. G. Hwang J. H. (2018). Biportal endoscopic decompression of exiting and traversing nerve roots through a single interlaminar window using a contralateral approach: technical feasibilities and morphometric changes of the lumbar canal and foramen. World Neurosurg. 117, 153–161. 10.1016/j.wneu.2018.05.111 29857220

[B2] An J. W. Lee C. W. (2019). Surgical treatment of extraforaminal gas-containing pseudocyst compressing L5 nerve root by using unilateral biportal endoscopy. World Neurosurg. 124, 145–150. 10.1016/j.wneu.2018.12.186 30659964

[B3] Barbiro-Michaely E. Mayevsky A. (2003). Effects of elevated ICP on brain function: can the multiparametric monitoring system detect the 'Cushing Response. Neurol. Res. 25 (1), 42–52. 10.1179/016164103101201102 12564125

[B4] Barry C. M. Van Den Heuvel C. Helps S. Vink R. (2012). Cushing’s mechanism maintains cerebral perfusion pressure in experimental subarachnoid haemorrhage. Neurosci. Lett. 529 (1), 92–96. 10.1016/j.neulet.2012.08.057 22982148

[B5] Choi D. J. Choi C. M. Jung J. T. Lee S. J. Kim Y. S. (2016). Learning curve associated with complications in biportal endoscopic spinal surgery: challenges and strategies. Asian Spine J. 10 (4), 624–629. 10.4184/asj.2016.10.4.624 27559440 PMC4995243

[B6] Dow D. E. Mantilla C. B. Zhan W. Z. Sieck G. C. (2006). EMG-based detection of inspiration in the rat diaphragm muscle. Conf. Proc. IEEE Eng. Med. Biol. Soc. 2006, 1204–1207. 10.1109/IEMBS.2006.260688 17946030

[B7] Gill J. Simopoulos T. Orhurhu V. Nagda J. Aner M. (2020). Lumbar epidural contrast spread patterns for the interlaminar approach: three-dimensional analysis using antero-posterior, lateral, and contralateral Oblique views. Pain Med. 21 (4), 747–756. 10.1093/pm/pnz256 31609385

[B8] Golder F. J. Mitchell G. S. (2005). Spinal synaptic enhancement with acute intermittent hypoxia improves respiratory function after chronic cervical spinal cord injury. J. Neurosci. 25 (11), 2925–2932. 10.1523/JNEUROSCI.0148-05.2005 15772352 PMC6725150

[B9] Golder F. J. Reier P. J. Davenport P. W. Bolser D. C. (1985). Cervical spinal cord injury alters the pattern of breathing in anesthetized rats. J. Appl. Physiol. 91 (6), 2451–2458. 10.1152/jappl.2001.91.6.2451 11717204

[B10] Heo D. H. Lee N. Park C. W. Kim H. S. Chung H. J. (2020). Endoscopic unilateral laminotomy with bilateral discectomy using biportal endoscopic approach: technical report and preliminary clinical results. World Neurosurg. 137, 31–37. 10.1016/j.wneu.2020.01.190 32028006

[B11] Heo D. H. Son S. K. Eum J. H. Park C. K. (2017). Fully endoscopic lumbar interbody fusion using a percutaneous unilateral biportal endoscopic technique: technical note and preliminary clinical results. Neurosurg. Focus 43 (2), E8. 10.3171/2017.5.FOCUS17146 28760038

[B12] Hong J. H. Jung S. W. Park J. H. (2017). Posture influences the extent of spread of contrast medium during thoracic epidurography: a prospective randomized trial. Pain Physician 20 (6), 501–508. 28934781

[B13] Hurst R. W. Kenyon L. C. Lavi E. Raps E. C. Marcotte P. (1995). Spinal dural arteriovenous fistula: the pathology of venous hypertensive myelopathy. Neurology 45 (7), 1309–1313. 10.1212/wnl.45.7.1309 7617189

[B14] Ito Z. Shibayama M. Nakamura S. Yamada M. Kawai M. Takeuchi M. (2021). Clinical comparison of unilateral biportal endoscopic laminectomy versus microendoscopic laminectomy for single-level laminectomy: a single-center, retrospective analysis. World Neurosurg. 148, e581–e588. 10.1016/j.wneu.2021.01.031 33476779

[B15] Jeong Y. C. Lee C. H. Kang S. Yoon J. S. (2017). Contrast spread in the superoposterior approach of transforaminal epidural steroid injections for lumbosacral radiculopathy. Ann. Rehabilitation Med. 41 (3), 413–420. 10.5535/arm.2017.41.3.413 PMC5532346 28758078

[B16] Jiang H. W. Chen C. D. Zhan B. S. Wang Y. L. Tang P. Jiang X. S. (2022). Unilateral biportal endoscopic discectomy versus percutaneous endoscopic lumbar discectomy in the treatment of lumbar disc herniation: a retrospective study. J. Orthop. Surg. Res. 17 (1), 30. 10.1186/s13018-022-02929-5 35033143 PMC8760683

[B17] Joh J. Y. Choi G. Kong B. J. Park H. S. Lee S. H. Chang S. H. (2009). Comparative study of neck pain in relation to increase of cervical epidural pressure during percutaneous endoscopic lumbar discectomy. Spine (Phila Pa 1976) 34 (19), 2033–2038. 10.1097/BRS.0b013e3181b20250 19675511

[B18] Kim J. E. Choi D. J. (2018). Biportal endoscopic transforaminal lumbar interbody fusion with arthroscopy. Clin. Orthop. Surg. 10 (2), 248–252. 10.4055/cios.2018.10.2.248 29854350 PMC5964275

[B19] Kim J. E. Choi D. J. Park E. J. (2020). Risk factors and options of management for an incidental dural tear in biportal endoscopic spine surgery. Asian Spine J. 14 (6), 790–800. 10.31616/asj.2019.0297 32429015 PMC7788375

[B20] Kim S. K. Kang S. S. Hong Y. H. Park S. W. Lee S. C. (2018). Clinical comparison of unilateral biportal endoscopic technique versus open microdiscectomy for single-level lumbar discectomy: a multicenter, retrospective analysis. J. Orthop. Surg. Res. 13 (1), 22. 10.1186/s13018-018-0725-1 29386033 PMC5793344

[B21] Kinoshita N. Tanaka S. Sugimori Y. Nakahira K. Ryoke K. Matsuoka T. (2018). High contrast between lumbar nerve roots and surrounding structures using dual echo 3D turbo spin echo additional fusion images. Jpn. J. Radiol. 36 (8), 472–476. 10.1007/s11604-018-0751-2 29948546

[B22] Kramer C. Jordan D. Kretschmer A. Lehmeyer V. Kellermann K. Schaller S. J. (2014). Electromyographic permutation entropy quantifies diaphragmatic denervation and reinnervation. PLoS One 9 (12), e115754. 10.1371/journal.pone.0115754 25532023 PMC4274091

[B23] Lee H. G. Kang M. S. Kim S. Y. Cho K. C. Na Y. C. Cho J. M. (2021). Dural injury in unilateral biportal endoscopic spinal surgery. Glob. Spine J. 11 (6), 845–851. 10.1177/2192568220941446 PMC8258823 32762357

[B24] Lewandrowski K. U. Hellinger S. De Carvalho P. S. T. Freitas Ramos M. R. Soriano-SáNchez J. A. Xifeng Z. (2021). Dural tears during lumbar spinal endoscopy: surgeon skill, training, incidence, risk factors, and management. Int. J. Spine Surg. 15 (2), 280–294. 10.14444/8038 33900986 PMC8059391

[B25] Mantilla C. B. Greising S. M. Zhan W. Z. Seven Y. B. Sieck G. C. (1985)2013). Prolonged C2 spinal hemisection-induced inactivity reduces diaphragm muscle specific force with modest, selective atrophy of type IIx and/or IIb fibers. J. Appl. Physiol. 114 (3), 380–386. 10.1152/japplphysiol.01122.2012 PMC3568873 23195635

[B26] Mantilla C. B. Seven Y. B. Hurtado-Palomino J. N. Zhan W. Z. Sieck G. C. (2011). Chronic assessment of diaphragm muscle EMG activity across motor behaviors. Respir. Physiol. Neurobiol. 177 (2), 176–182. 10.1016/j.resp.2011.03.011 21414423 PMC3103648

[B27] Pásztor A. Pásztor E. (1980). Spinal vasomotor reflex and Cushing response. Acta Neurochir. (Wien) 52 (1-2), 85–97. 10.1007/BF01400952 7376951

[B28] Shi B. Li X. Li H. Ding Z. (2012). The morphology and clinical significance of the dorsal meningovertebra ligaments in the lumbosacral epidural space. Spine (Phila Pa 1976) 37 (18), E1093–E1098. 10.1097/BRS.0b013e31825c05ea 22565391

[B29] Soliman H. M. (2013). Irrigation endoscopic discectomy: a novel percutaneous approach for lumbar disc prolapse. Eur. Spine J. 22 (5), 1037–1044. 10.1007/s00586-013-2701-0 23392557 PMC3657046

[B30] Soliman H. M. (2015). Irrigation endoscopic decompressive laminotomy. A new endoscopic approach for spinal stenosis decompression. Spine J. 15 (10), 2282–2289. 10.1016/j.spinee.2015.07.009 26165475

[B31] Soliman H. M. (2016). Irrigation endoscopic assisted percutaneous pars repair: technical note. Spine J. 16 (10), 1276–1281. 10.1016/j.spinee.2016.06.009 27345745

[B32] Stepińska G. Czernicki Z. Berdyga J. Jurkiewiz J. (1995). Transcranial Doppler sonography in experimental Cushing response. Acta Neurochir. (Wien) 133 (1-2), 80–82. 10.1007/BF01404953 8561043

[B33] Uchikado H. Nishimura Y. Hattori G. Ohara Y. (2020). Micro-anatomical structures of the lumbar intervertebral foramen for full-endoscopic spine surgery: review of the literature. J. Spine Surg. 6 (2), 405–414. 10.21037/jss.2019.10.07 32656378 PMC7340827

[B34] Vesovski S. Makara M. Martinez-Taboada F. (2019). Computer tomographic comparison of cranial spread of contrast in lumbosacral and sacrococcygeal epidural injections in dog cadavers. Vet. Anaesth. Analg. 46 (4), 510–515. 10.1016/j.vaa.2019.02.007 31155379

[B35] Vinit S. Stamegna J. C. Boulenguez P. Gauthier P. Kastner A. (2007). Restorative respiratory pathways after partial cervical spinal cord injury: role of ipsilateral phrenic afferents. Eur. J. Neurosci. 25 (12), 3551–3560. 10.1111/j.1460-9568.2007.05619.x 17610574

[B36] Warren P. M. Steiger S. C. Dick T. E. MacFarlane P. M. Alilain W. J. Silver J. (2018). Rapid and robust restoration of breathing long after spinal cord injury. Nat. Commun. 9 (1), 4843. 10.1038/s41467-018-06937-0 30482901 PMC6258702

